# Experimental Research on Rapid Localization of Acoustic Source in a Cylindrical Shell Structure without Knowledge of the Velocity Profile

**DOI:** 10.3390/s21020511

**Published:** 2021-01-13

**Authors:** Jia Fu, Shenxin Yin, Zhiwen Cui, Tribikram Kundu

**Affiliations:** 1 Department of Acoustics and Microwave Physics, College of Physics, Jilin University, Changchun 130012, China; fujia17@mails.jlu.edu.cn; 2 College of Aerospace Engineering, Chongqing University, Chongqing 400044, China; shenxinyin@cqu.edu.cn; 3 Department of Civil and Architectural Engineering and Mechanics, University of Arizona, Tucson, AZ 85721, USA; tkundu@email.arizona.edu; 4 State Key Laboratory of Acoustics, Institute of Acoustics, Chinese Academy of Sciences, Beijing 100190, China; 5 Aerospace and Mechanical Engineering Department, University of Arizona, Tucson, AZ 85721, USA

**Keywords:** acoustic emission, acoustic source localization, cylindrical vessel, the time difference of arrival, arbitrary triangle time difference technology

## Abstract

Acoustic source localization in a large pressure vessel or a storage tank-type cylindrical structure is important in preventing structural failure. However, this can be challenging, especially for cylindrical pressure vessels and tanks that are made of anisotropic materials. The large area of the cylindrical structure often requires a substantial number of sensors to locate the acoustic source. This paper first applies conventional acoustic source localization techniques developed for the isotropic, flat plate-type structures to cylindrical structures. The experimental results show that the conventional acoustic source localization technique is not very accurate for source localization on cylindrical container surfaces. Then, the L-shaped sensor cluster technique is applied to the cylindrical surface of the pressure vessel, and the experimental results prove the applicability of using this technique. Finally, the arbitrary triangle-shaped sensor clusters are attached to the surface of the cylindrical structure to locate the acoustic source. The experimental results show that the two acoustic source localization techniques using sensor clusters can be used to monitor the location of acoustic sources on the surface of anisotropic cylindrical vessels, using a small number of sensors. The arbitrarily triangle-shaped sensors can be arbitrarily placed in a cluster on the surface of the cylindrical vessel. The results presented in this paper provide a theoretical and experimental basis for the surface acoustic source localization method for a cylindrical pressure vessel and lay a theoretical foundation for its application.

## 1. Introduction

Acoustic source localization (ASL) technology plays an indispensable role in the application of nondestructive testing (NDT) and structural health monitoring (SHM) [1]. Materials are prone to cracks due to repeated loading that causes fatigue in materials and gives rise to fatigue cracks. Crack initiation and propagation generate acoustic signals [2]. By locating these harmful acoustic emission sources, the points of initiation or localization of cracks or damages can be identified well before the failure of the structure. Therefore, ASL can effectively reduce the economic losses by increasing the safety of structures. 

Cylindrical vessels and containers use less material than many other shapes for a vessel with the same volume and thickness. In addition, the cylindrical vessel is relatively easy to make, relatively strong, and not easily damaged by the liquid in the vessel by avoiding regions of high-stress concentration. Therefore, for convenience in manufacturing and ease of transportation, cylindrical containers are widely used for storage and transportation. The huge potential for safety hazards, due to undetected damages, has made the structural health of cylindrical pressure vessels and tanks a perpetual concern for researchers. SHM strategies based on acoustic emissions (AE) have been widely used [3,4,5,6,7,8,9,10,11] over the last several years. Traditional techniques work very well when the wave velocity variation in a cylindrical structure is low, the structure can be assumed to be isotropic, and the arrival times of the waves at all sensor locations are known [12]. The surface of the cylindrical structure when unfolded along the generatrix is a two-dimensional curved rectangular plate [13]. The bending needed to form the cylindrical structure makes the velocity distribution anisotropic, or, in other words, no longer uniform. Traditional ASL techniques that do not consider the anisotropic properties of the material give incorrect predictions [14]. Asty [15] proposed a method to locate the seismic source on a spherical surface using a proper coordinate system. This method locates the acoustic emission source from the time difference between the acoustic source and each sensor. Yoon et al. [16] used the path difference of several propagation directions of the acoustic emission source in a cylindrical structure to locate the acoustic emission source. Investigations that consider the anisotropic properties of cylindrical structures are relatively few in number. An objective function-based technique, developed by Hajzargerbashi et al. [17], used the cylindrical coordinates of four sensors attached to the cylinder. Four arrival times at these four sensors were used to locate the point of impact by minimizing the objective function, which is the least squares error expression. Nakatani et al. [18] applied a beamforming array technique with four sensors to a cylindrical geometry to detect the point of impact. However, these techniques required the velocity distribution information in advance for the anisotropic structure. Therefore, it will be important to study the ASL technique whilst considering the anisotropic nature of the cylindrical structures, without a priori knowledge of the direction-dependent velocities.

A new time difference of arrival (TDOA)-based localization technique proposed by Kundu et al. [19,20] works well for isotropic and anisotropic plates without knowledge of the plate properties. Kundu’s technique places three sensors in an L-shape to predict the location of the acoustic source. This technique can be used for complex inhomogeneous structures [21], highly anisotropic structures [22], and three-dimensional structures [23]. Wu et al. [24] and Yin et al. [25] suggested various sensor placement alternatives in cluster geometries to achieve more flexibility and/or more accurate predictions. Niri et al. [14] attached the sensor to the surface of a cylindrical structure and used the probability theory to estimate the position of the acoustic source. Many scholars estimated the direction of arrival of guided waves from the TDOA information of sensor clusters [26,27,28,29,30]. In our previous work [31], the L-shaped sensor cluster method developed by Kundu et al. [15] was applied to the cylindrical pressure vessel wall. The preliminary experimental results showed that this method works equally well for flat and cylindrical plate geometries. Previously published works, e.g., [20,24], were only applicable to plate structures. This paper extends the acoustic source localization techniques developed for flat plate-type structures to cylindrical structures and verifies the technique experimentally. As an extended work of [31], this paper will use L-shaped sensor clusters for source localization in an anisotropic cylindrical vessel. Arbitrary sensor array geometry is also used to conduct experimental exploration on the cylindrical vessel at the same time, as this was not considered in [31]. The experimental results verify that these two methods can reliably predict the acoustic source position on the surface of a cylindrical structure, without a priori knowledge of the wave speed in that structure. It provides a new, alternative technique for ASL on cylindrical pressure vessels and containers.

## 2. Formulation

### 2.1. L-Shaped Sensor Cluster and Time Difference Positioning Technique

The surface of the cylindrical vessel unfolded along the generatrix is a two-dimensional curved rectangular plate. The shortest acoustic path of acoustic waves propagating between two points on the surface of a cylinder vessel is the straight line distance between the two points on the plane when the side of the cylinder is expanded [13]. 

**Figure 1:**
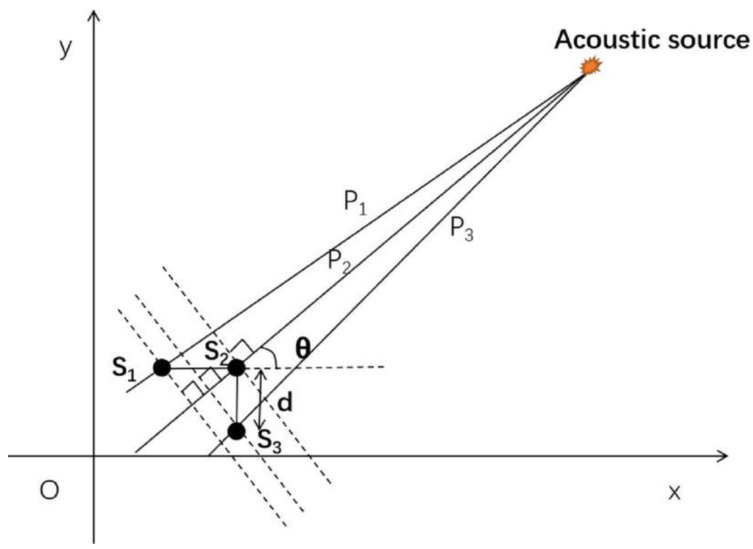
Schematic diagram of acoustic source localization by L-shaped time difference positioning technology.

**Figure 2:**
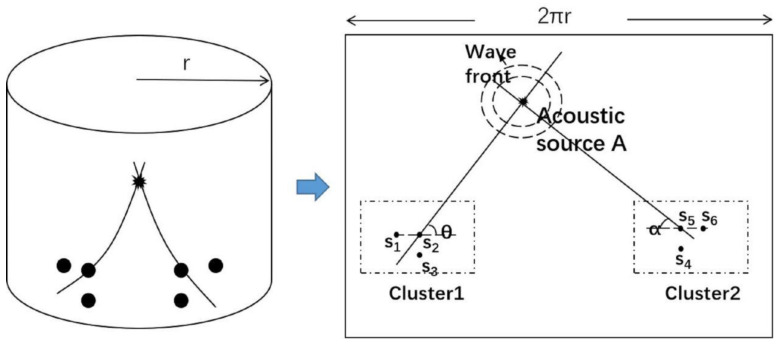
Schematic diagram of the L-shaped time difference positioning technique with two sets of sensors.

When the flat panel displays anisotropic properties, the L-shaped time difference positioning technique is illustrated in Figure 1 and Figure 2. The six sensors used are denoted s_1_–s_6_ and are divided into two sensor clusters, shown in Figure 2. In each cluster, three sensors are placed in a right-angled isosceles triangle. The coordinates of s_1_–s_3_ in sensor cluster one are (*x*_1_, *y*_1_), (*x*_2_, *y*_2_), and (*x*_3_, *y*_3_), and the acoustic source coordinate A is (*x*, *y*). The distances between the acoustic source and the sensor are *p*_1_, *p*_2_, and *p*_3_. The distance *d* between the sensors s_1_ and s_2_ and between s_2_ and s_3_ is the same. The distance between the acoustic source and any sensor in the cluster (*p*_1_, *p*_2_, or *p*_3_) is much greater than the distance *d* between the sensors. Then, the wave velocity *c* of the acoustic wave propagating from the acoustic source to the three sensors in the cluster can be assumed to be the same. The distance from the sensor to the acoustic source can be expressed as:

(1) $$p_{1}=c\times \left(t_{1}-t_{0}\right)$$

(2) $$p_{2}=c\times \left(t_{2}-t_{0}\right)$$

In the above formula, *t*_0_ is acoustic emission signal generation time. *t*_2_ is the wave travel time from the acoustic emission source to the sensor s_2._ The acoustic emission signal generation time (*t*_0_) cannot be obtained; hence, Equations (1) and (2) are combined to remove *t*_0_:

(3) $$p_{1}-p_{2}=c\times \left(t_{1}-t_{2}\right)=c\times t_{ij}$$

*t_ij_* is the time difference between the two sensors. The distance *d* between sensors can also be used to obtain the following equations:

(4) $$d\times \cos\theta =p_{1}-p_{2}=c\times \left(t_{1}-t_{2}\right)$$

(5) $$d\times \sin\theta =p_{3}-p_{2}=c\times \left(t_{3}-t_{2}\right)$$

The wave velocity of the acoustic wave propagating from the acoustic source to different sensors should be different, in general, in an anisotropic medium. However, since the distance between sensors in a cluster is far less than the distance between the sensor cluster and the AE source, the wave velocity from the source to the three sensors in a cluster can be assumed to be the same. The acoustic wave propagation direction can be eliminated by the following formula:

(6) $$\tan\theta =\frac{p_{3}-p_{2}}{p_{1}-p_{2}}=\frac{t_{12}}{t_{32}}=\frac{y-y_{2}}{x-x_{2}}$$

Similarly, the angle of another group of sensors can be expressed as:

(7) $$\tan\alpha =\frac{t_{65}}{t_{45}}=\frac{y-y_{5}}{x-x_{5}}$$

From Equations (6) and (7), two angles are obtained from the TDOA (time difference of arrival) values *t_ij_*. With these angles, the acoustic source position (*x*, *y*) can be obtained, as illustrated in Figure 2.

### 2.2. Arbitrary Triangular Cluster for a New Time Difference Positioning Technique

An L-shaped sensor cluster requires that the shape of the sensor cluster must be an isosceles right-angled triangle. This strict restriction on sensor placement requirement can be waived by adopting the work of Wu et al. [24] on granite slabs. A brief description of their derivation is presented below. 

**Figure 3:**
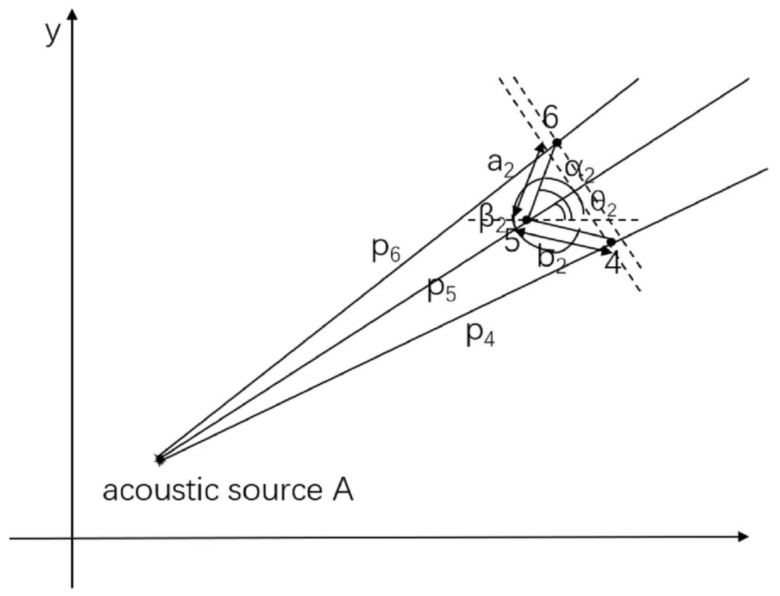
Schematic diagram of acoustic source localization for the arbitrary triangular-shaped cluster.

The method is applied to arrange the three sensors into an arbitrary triangle in order to quickly and accurately locate the acoustic source direction, as shown in Figure 3. The distance between sensors 4 and 5 is *b*_2_, and the distance between sensors 5 and 6 is *a*_2_. The distance from the acoustic source to sensor 4 is *P*_4_, the distance from sensor 5 is *P*_5_, and the distance from sensor 6 is *P*_6_. The angle between sensors 4 and 5 and the positive *x*-axis direction is *β*_2_, and the angle between sensors 5 and 6 and the positive *x*-axis direction is *α*_2_. The angle between the acoustic source to the sensor and the positive horizontal direction is *θ*_2_. The propagation distance of acoustic waves between sensors 5 and 6 can be expressed as

(8) $$P_{6}-P_{5}=a_{2}\cos\left(\alpha_{2}-\theta_{2}\right)=c_{2}\times t_{65}$$

The propagation distance of the acoustic wave between sensors 4 and 5 can be expressed as

(9) $$P_{4}-P_{5}=b_{2}\cos\left(\theta_{2}-\beta_{2}+2\pi \right)=c_{2}\times t_{45}$$

**Figure 4:**
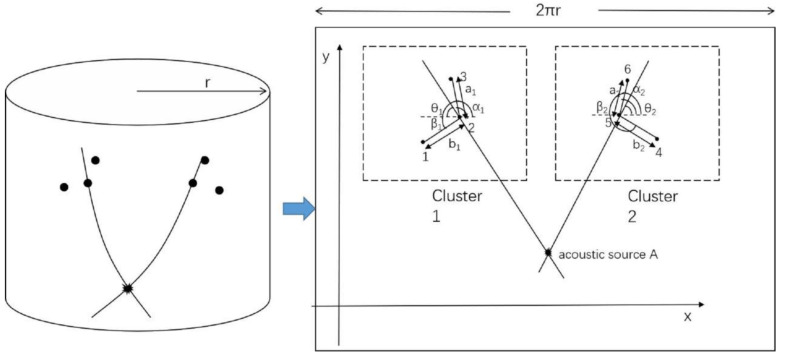
Schematic diagram of arbitrary triangular-shaped clusters for two sets of sensors.

As the distance from the acoustic source to the sensor cluster is much greater than the distance between the sensors, it can be assumed that the wave speed is the same for all sensors in a cluster. The acoustic wave speeds from the acoustic source to the sensor clusters 1 and 2 shown in Figure 4 are denoted as *c*_1_ and *c*_2_, respectively. From Equation (8) and (9), we obtain:

(10) $$\frac{b_{2}\cos\left(\theta_{2}-\beta_{2}\right)}{a_{2}\cos\left(\alpha_{2}-\theta_{2}\right)}=\frac{b_{2}}{a_{2}}\frac{\cos\beta_{2}-\tan\theta_{2}\sin\beta_{2}}{\cos\alpha_{2}+\tan\theta_{2}\sin\alpha_{2}}=\frac{t_{45}}{t_{65}}$$

Therefore, for sensor cluster 2

(11) $$\tan\theta_{2}=\frac{y_{5}-y}{x_{5}-x}=\frac{b_{2}t_{65}\cos\beta_{2}-a_{2}t_{45}\cos\alpha_{2}}{a_{2}t_{45}\sin\alpha_{2}-b_{2}t_{65}\sin\beta_{2}}$$

Similarly, the propagation direction for sensor cluster 1 can be obtained:

(12) $$\tan\theta_{1}=\frac{y_{2}-y}{x_{2}-x}=\frac{b_{1}t_{32}\cos\beta_{1}-a_{1}t_{12}\cos\alpha_{1}}{a_{1}t_{12}\sin\alpha_{1}-b_{1}t_{32}\sin\beta_{1}}$$

The point of intersection of the directions, thus derived from the two clusters of sensors, gives the acoustic source position. Therefore, the time information obtained experimentally can be substituted in the above equation to calculate the acoustic source coordinates.

The experimental error of acoustic source localization in two-dimensional plate structures can be calculated using the following formula:

(13) $$e=\sqrt{{\left(x-x_{A}\right)}^{2}+{\left(y-y_{A}\right)}^{2}}$$

## 3. Experimental Investigation

The side wall of a cylindrical vessel was used to conduct the acoustic emission experiment in the laboratory. The side wall dimensions of the cylindrical structure were 1 meter in diameter, 1 meter in height, and 3 mm in thickness. The radius of the main container of this part of the side wall was 0.5 m. In the absence of a multichannel oscilloscope, the experiment was conducted using simple instruments to verify the theory. The experiment used an Agilent oscilloscope, a single-channel ultrasonic transceiver system, and two 150 kHz ultrasonic sensors. The frequency range of the small acoustic emission sensor we selected was 60–400 kHz, and the resonance frequency was 150 kHz. This frequency covers the frequency range of most acoustic emission events. This acoustic emission sensor is suitable for metals used for pressure vessels. One of the two sensors was used to excite the acoustic signal, and the other was used to receive the signal. The diameter of the ultrasonic sensor was 1.4 cm.

**Figure 5:**
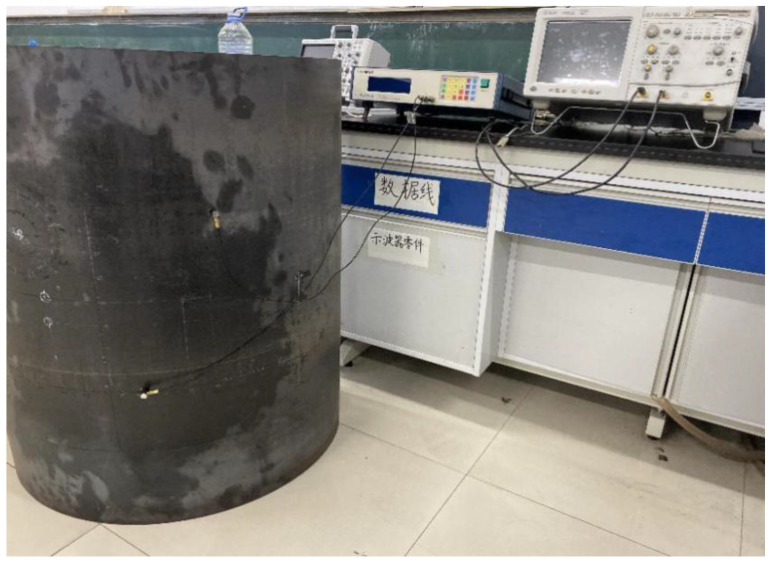
Experimental system. The experiment only uses two sensors to achieve acoustic source localization. One of the sensors is placed at the acoustic source position, and the second one at the receiver positions, marked before the experiment.

**Figure 6:**
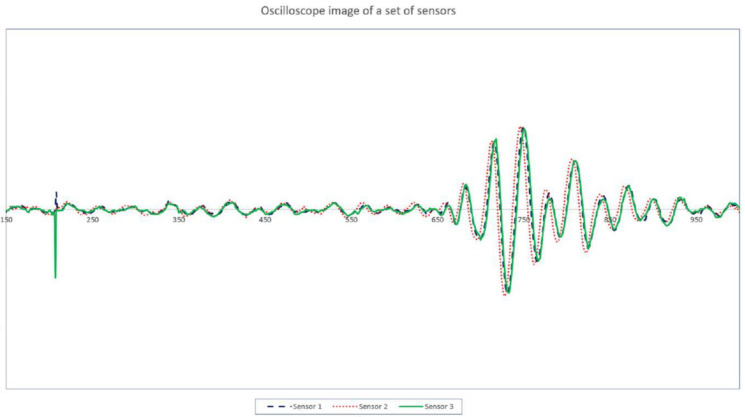
The waveforms received by the sensor at three different positions (S1, S2, and S3).

The experimental setup is shown in Figure 5. A cylindrical coordinate system was established on the surface of the vessel, with the center of the vessel as the coordinate origin. The unit of the coordinate system was 1 cm. For the single-channel ultrasonic transceiver system in the laboratory, one of the ultrasonic transducers was placed in a fixed position to simulate the acoustic source. The other one, in turn, was placed from the S1 to S6 positions to receive the acoustic signal, which is used to obtain the arrival time information at the receiving sensor. For example, the waveforms received by the sensor at three different positions (S1, S2, S3) are shown in Figure 6. The peak of the first waveform was chosen for the timing point. Even if we do not know the exact time of the acoustic event, this time can be eliminated by taking the time difference of arrival times at the sensors in the group, as shown in Equation (3). If one has a multichannel oscilloscope in the laboratory, then several sensors can be used simultaneously, and the arrival time information of the 6 sensors can be obtained at one time.

Since the acoustic properties of the containment wall material are unknown, we must first assume that the containment wall material is anisotropic. In the experiment, an isotropic localization method was selected to predict the acoustic source. First, the conventional triangulation technique was chosen. The acoustic source coordinates were randomly selected from 5 locations. The cylindrical coordinates of the sensor used to receive the signal were (0, 0), (20, 0), and (10, 20). In the experiment, one sensor was used to simulate the acoustic source, and the other sensor was used to receive the acoustic signal. Acoustic waves travel along the shortest path in the medium. In the sensor cluster, the peak arrival time was obtained from the first arrival waveform received by each sensor. The experimental data were fed into the equations derived above to obtain the acoustic source position. It is important to remember that, during the experimental measurement, there may be some error in determining when the sensor receives the first wave. Hence, we repeated every set of experiments 10 times to reduce the error. Finally, the average value received by each sensor was considered for the final calculation. Table 1 shows the acoustic source locations predicted by the conventional triangulation technique [32]. Equation (13) calculates the experimental error. The coordinates (x, y) are the predicted acoustic source coordinates on the side wall, and (x_A_, y_A_) are the actual acoustic source coordinates. Wu et al. [24] and Yin et al. [25] previously verified the theoretical method experimentally. The area they had selected for the experiment was 50 × 50 cm. Their prediction error was about 2 cm (average distance between the true position and the predicted position of the acoustic source). 

The positioning results in the table are converted from plane coordinates to cylindrical coordinates (r, θ, z). As the radius was a constant at 0.5m, we only show (θ, z) in the following tables. The experimental error represents the distance between the true position and the predicted position of the acoustic source. From the experimental results, it can be concluded that the average error of the traditional triangulation technique was 4.77 cm.

Next, we localized the acoustic emission source from the diamond-shaped array on the surface of the vessel [2]. The experimental method here is the same as the conventional triangulation technique. Here, an additional sensor was used. The coordinates of the four receiving sensors were (0, 10), (10, 0), (20, 10), and (10, 20). This requires the measured times at the four receiving sensors to be substituted in the source localization algorithm to calculate the acoustic source position. The predictions of this experiment are listed in Table 2. The average error was equal to 4.16 cm.

In order to make the experimental results more accurate, the number of receiving sensors was then increased to six. The acoustic source was predicted by minimizing the error function [20]. The coordinates of the receiving sensors were (0, 10), (0, 0), (10, 0), (20, 10), (20, 20), and (10, 20). For the acoustic source, five positions were randomly selected. The position of the acoustic source was predicted by substituting the time received at the sensor position into the error function and minimizing it. The experimental results are shown in Table 3. The average error of the experimental results was 2.90 cm.

**Figure 7:**
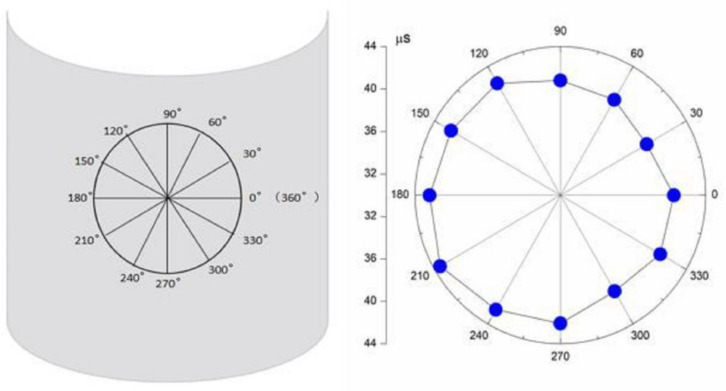
Arrival times in different directions with 30° increments. The receiver is placed on a circle of radius 8 cm.

Since the experimental results did not significantly improve when the number of sensors is increased from 3 to 6, one can conclude that the initial assumption of the material being isotropic may not be correct. Hence, the acoustic velocity distribution on the sidewall was measured. The arrival time was measured for every 30° interval at a distance of 8 cm from the acoustic source. The arrival time distribution as a function of the propagation direction measured in the laboratory is shown in Figure 7. It can be concluded from the experimental data that the acoustic velocity distribution on the surface of the vessel wall is not uniform. Instead, it is weakly anisotropic. As both the L-shaped sensor clusters and arbitrary triangular-shaped clusters are capable of acoustic source localization in anisotropic structures, experiments with these sensor clusters were conducted.

**Figure 8:**
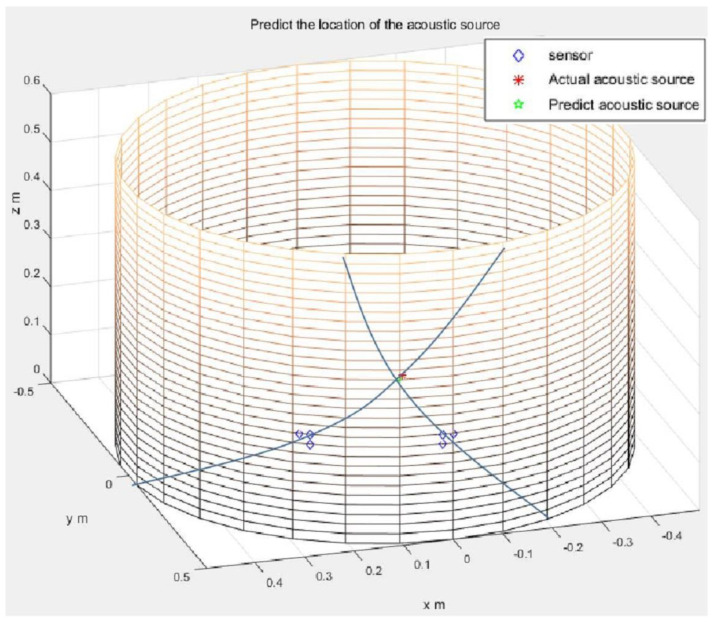
The actual acoustic source location (denoted by the asterisk) and the predicted acoustic source location (intersection points of the two lines, obtained from L-shaped sensor clusters) for the cylindrical structure.

For the L-shaped cluster arrangement proposed by Kundu [20], the coordinate axis on the side wall of the cylindrical vessel was marked. The acoustic source and sensor cluster positions were randomly selected. Within a cluster, the distance between two sensors in two orthogonal directions is taken as *d* = 2 cm. The time difference of arrival *t_ij_* is obtained by subtracting the peak time of the waveform collected by each sensor. The acoustic source position obtained from this time difference information is shown in Figure 8. It can be seen in this figure that the two straight lines are the directions from the two clusters of sensors to the acoustic source, and the intersection point is the acoustic source position. Nine acoustic source positions were randomly selected for this experiment, and all of the experimental results are listed in Table 4. The actual acoustic source position and the measured acoustic source position are compared in the table. The experimental errors are shown in the last column.

**Figure 9:**
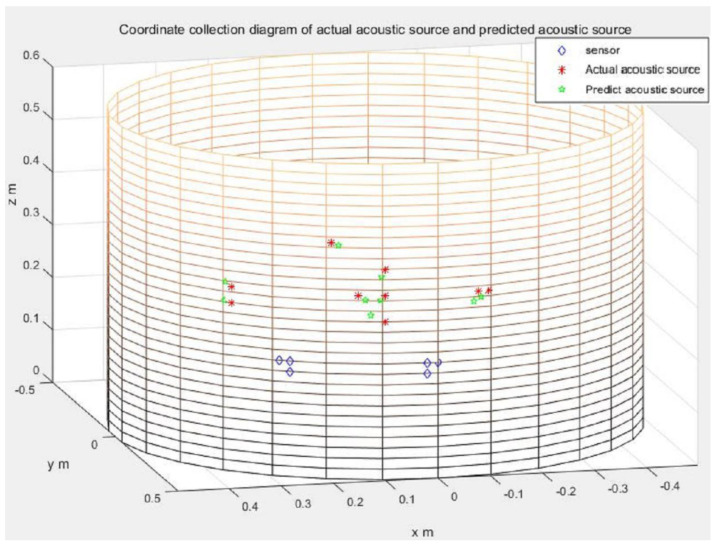
Experimental results: the asterisks denote the actual positions of the acoustic source, the pentagons denote the predicted positions of the acoustic source, and the diamonds are the receiving sensor positions. (for L-shaped sensor clusters).

In Figure 9, we show both the actual and the predicted acoustic source coordinates for easy comparison. This diagram demonstrates that the experimental results show that L-shaped sensor clusters can accurately localize the acoustic source locations on the side wall of the cylindrical vessel. 

**Figure 10:**
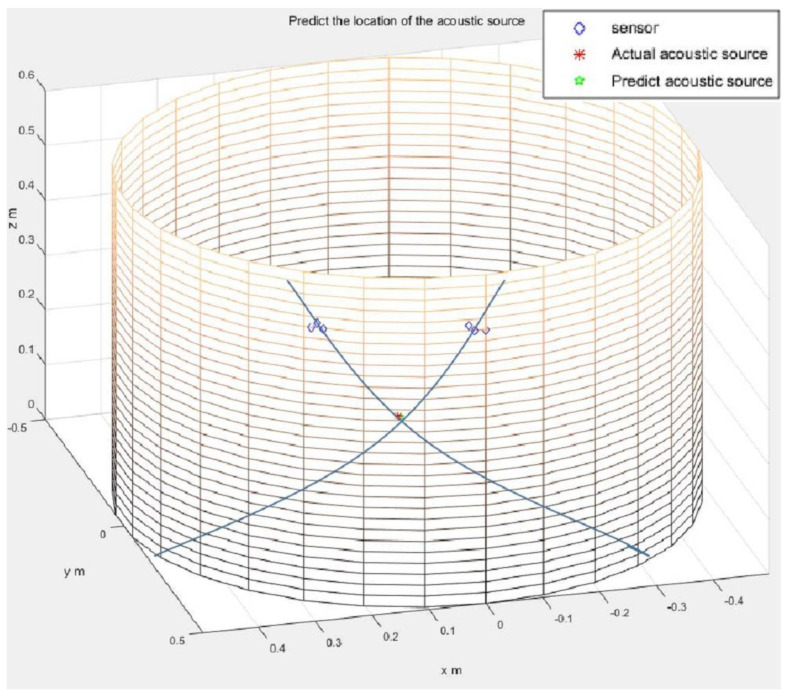
The actual acoustic source location (denoted by the asterisk) and the predicted acoustic source location (intersection points of the two lines, obtained from arbitrary triangular-shaped sensor clusters) for the cylindrical structure.

**Figure 11:**
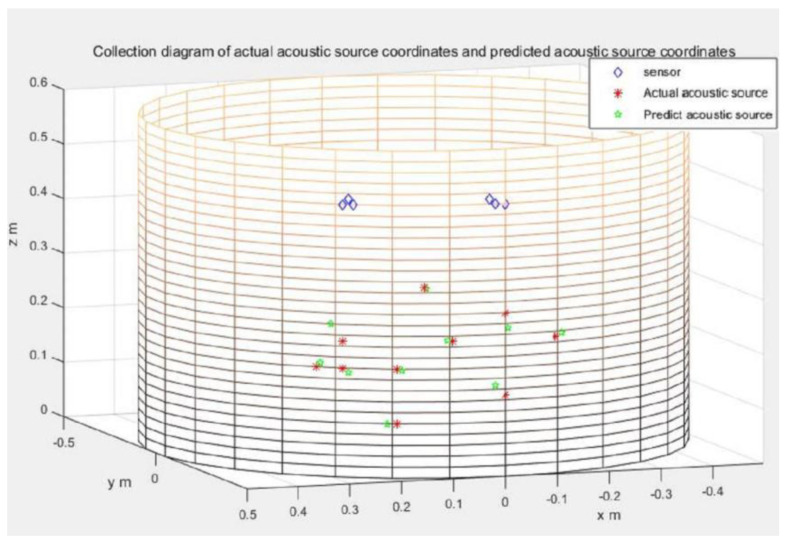
Experimental results: the asterisks denote the actual positions of the acoustic source, the pentagons denote the predicted positions of the acoustic source, and the diamonds are the receiving sensor positions. (for arbitrary triangular-shaped sensor clusters).

The specimen used for the experimental investigation was a cylindrical pressure vessel side wall, as shown in Figure 5, Figure 6, Figure 7, Figure 8 and Figure 9. It was necessary to make sure that the two arms of the L-shaped sensor cluster were exactly orthogonal to each other. To waive this strict restriction, we used the arbitrary triangular-shaped sensor cluster proposed by Wu et al. [24] to localize the acoustic source. The instruments and conduct methods used in the experiment were the same as those used to calculate the time difference of the arrival method. We then randomly selected the acoustic source position and placed a sensor at the acoustic source position. The other sensor was placed at the selected sensor position to receive the signal. The sensor coordinates used to receive the signal were (1, 40), (2, 40), (3, 41), (28, 40), (29, 41), and (30, 40). The experimental data on arrival times were then substituted using the appropriate equations to localize the acoustic source, as shown in Figure 10. Ten sets of source locations were selected for the experiment. The signals collected by the receiving sensors for every acoustic source position were measured 10 times, and the average value was taken to reduce experimental errors. The experimental results are shown in Table 5. The actual acoustic source position and the measured acoustic source position are compared in the table, and the errors in prediction are also shown. In order to observe the experimental results more clearly, the actual and the predicted acoustic source coordinates are shown together for easy comparison in Figure 11. The average error of the experimental results was 1.77 cm.

From the experimental results, it can be observed that the average error of the arbitrary triangular time difference technique is 1.69 cm. The experimental results also show that the average error of the L-shaped time difference positioning technique and arbitrary triangular-shaped sensor clusters is smaller than the localization method, which only applies to the isotropic case, as used above (conventional triangulation technique, diamond-shaped sensor array configuration, and six-sensor positioning method).

The assumption that the circular wavefront can be regarded as a plane wavefront when the distance between the sensor and the sound source is much larger than the distance between the sensors may introduce some error when the distance between the sensor cluster and the acoustic source is not very large. Some experimental errors may also be introduced from sensor positioning error from one experiment to another.

## 4. Conclusions

In this experiment, we used several acoustic source localization techniques developed for flat plates and investigated their applicability potential for source localization in cylindrical structures. The experimental results show that these acoustic source localization techniques can also localize the acoustic source on the surface of the cylindrical vessel wall. The number of sensors used for both methods in this paper is less than the number of sensors used in sensor array-based monitoring methods. At present, a large number of sensor array positioning methods are used in industry for cylindrical shell structures. The localization method used in this paper does not need to solve complex nonlinear equations. The solution of linear equations gives rise to faster localization. Thus, the arbitrary triangular-shaped and L-shaped sensor clusters have the advantages of using fewer sensors, requiring less calculation and, hence, higher processing speed. It also does not need the information on acoustic velocity distribution in the structure. Arbitrary triangular-shaped clusters also require a total of six sensors, like L-shaped clusters, to localize an acoustic source, and they have fewer restrictions on the sensor placement requirements. The two sensor cluster-based methods presented in this paper have a higher potential for in situ monitoring of acoustic sources in cylindrical pressure vessels and containers when compared to other conventional techniques.

## Figures and Tables

**Table 1:** Acoustic source localization results from the conventional triangulation technique.

No.	Exact Position (θ, z/cm)	Predicted Position (θ, z/cm)	Error/cm
1	(11.45°, 10)	(11.50°, 6.46)	3.54
2	(8.02°, 7)	(11.41°, 7.47)	3.00
3	(12.60°, 5)	(11.47°, 7.92)	3.06
4	(6.87°, 2)	(11.37°, 7.48)	6.71
5	(11.45°, 15)	(11.55°, 7.45)	7.55

**Table 2:** Experimental results for the diamond-shaped sensor array configuration.

No.	Exact Position (θ, z/cm)	Predicted Position (θ, z/cm)	Error/cm
1	(11.45°, 10)	(11.59°, 6.57)	3.43
2	(8.02°, 7)	(11.29°, 7.81)	2.97
3	(12.60°, 5)	(11.17°, 7.83)	3.09
4	(6.87°, 2)	(10.82°, 7.19)	6.23
5	(11.45°, 15)	(12.74°,10.03)	5.09

**Table 3:** Experimental results using six sensor positioning methods.

No.	Exact Position (θ, z/cm)	Predicted Position (θ, z/cm)	Error/cm
1	(11.45°, 10)	(8.82°, 8.6)	2.69
2	(8.02°, 7)	(7.39°, 9.24)	2.31
3	(12.60°, 5)	(12.31°, 7.53)	2.53
4	(6.87°, 2)	(11.17°, 0.075)	4.21
5	(11.45°, 15)	(8.82°, 16.5)	2.74

**Table 4:** Acoustic source localization experimental results for L-shaped sensor clusters.

No.	Exact Position (θ, z/cm)	Predicted Position (θ, z/cm)	Error/cm
1	(11.45°, 15)	(12.26°, 14.10)	1.14
2	(−11.45°, 15)	(−9.72°, 13.99)	1.81
3	(17.18°, 15)	(15.52°,14.06)	1.73
4	(45.83°, 15)	(47.39°, 15.97)	1.67
5	(22.91°, 25)	(21.31°, 24.39)	1.53
6	(11.45°, 10)	(14.47°, 11.29)	2.93
7	(11.45°, 20)	(12.18°,18.59)	1.54
8	(−9.16°, 15)	(−8.02°, 13.30)	1.97
9	(45.83°, 12)	(47.67°, 12.36)	1.64

**Table 5:** Acoustic source localization experimental results for arbitrary triangular-shaped sensor clusters.

No.	Exact Position (θ, z/cm)	Predicted Position (θ, z/cm)	Error/cm
1	(17.18°, 25)	(16.76°, 24.81)	0.42
2	(−11.45°, 15)	(−13.07°, 15.61)	1.54
3	(34.37°, 10)	(33.30°, 9.27)	1.19
4	(22.91°, 10)	(21.88°, 9.83)	0.92
5	(34.37°, 15)	(36.95°, 17.97)	3.73
6	(22.91°, 0)	(25.01°, −0.06)	1.83
7	(40.10°, 10)	(39.31°, 10.76)	1.03
8	(0°, 20)	(−0.48°, 17.29)	2.74
9	(0°, 5)	(2.14°, 6.76)	2.57
10	(11.45°, 15)	(12.44°, 15.20)	0.88

## Data Availability

Data sharing is not applicable to this article.
